# The Effects of Onychectomy (Declawing) on Forearm and Leg Myology in a Kinkajou (*Potos flavus*)

**DOI:** 10.3390/ani14192774

**Published:** 2024-09-26

**Authors:** Lara L. Martens, Reece A. Brown, Ana Carolina Lourenço Faillace, Arin Berger, Rachel L. J. Smith, Kathryn Bertok, Lauren Humphries, Angela Lassiter, Adam Hartstone-Rose

**Affiliations:** 1 Department of Biological Sciences, North Carolina State University, Raleigh, NC 27695, USA; llmarten@ncsu.edu (L.L.M.); rabrow26@ncsu.edu (R.A.B.); ana.faillace@aluno.unb.br (A.C.L.F.); snberger@ncsu.edu (A.B.); rlsmit11@ncsu.edu (R.L.J.S.); 2 Wild Animal Anatomy Research Laboratory, School of Agricultural Science and Veterinary Medicine, University of Brasília, Brasília CEP 70636-200, Brazil; 3 Carolina Tiger Rescue, Pittsboro, NC 27312, USA; kathrynbertok@carolinatigerrescue.org (K.B.); laurenhumphries@carolinatigerrescue.org (L.H.); angelalassiter@carolinatigerrescue.org (A.L.)

**Keywords:** *Potos flavus*, antebrachial muscles, crural muscles, physiological cross-sectional area, fascicles, muscle architecture, Procyonidae

## Abstract

**Simple Summary:**

“Declawing” is the surgery in which the bone underneath the claw is removed entirely or in part. This has been shown to have substantial effects on the forearm muscles of members of the cat family, but no one has previously examined how it affects other species or the hindlimb. In this study, we examine the leg and forearm muscles of a kinkajou (a Central/South American tree-climbing relative of the raccoon) that was declawed on all four limbs and compare it to several kinkajous that were not declawed and to the previous cat findings. As expected, some of the declawed kinkajou’s muscles were substantially different from those of the intact specimens, and as was seen in the cats, the muscles that normally attach to its claw bones appear to have been weaker. Surprisingly, the declawed kinkajou had larger forearm muscles and, even though its toe claws had also been removed, its hindlimb muscles were not very different—possibly because kinkajous rely more on their hands than their feet. Future studies should examine other declawed kinkajous and how this surgery affects other species, like kinkajou relatives that don’t climb as much or other species, like monkeys, that do climb like kinkajous.

**Abstract:**

Recently, onychectomy, the “declaw” surgery in which all or part of the distal phalanges are removed, has been shown to have significant effects on the forearm muscles of felids. While this surgery should clearly affect the limb muscles (especially those that insert on the removed or modified bone), these effects have not been studied beyond felids or in the hindlimb. To that end, we herein evaluated the muscle architecture of a kinkajou (*Potos flavus*) that was declawed on all four of its limbs and compared its anatomy to that of intact specimens and the felid findings. As expected, some of the declawed kinkajou’s muscles were substantially different from those of the intact specimens, and as was seen in felids, its digital muscles appear to have been weaker. However, unlike in the felids, the declawed kinkajou had relatively larger forearm muscles. Also, contrary to expectation, the leg muscles of the declawed kinkajou were not substantially different, perhaps reflecting important differences in limb use. Future analyses should examine this anatomy in other declawed kinkajou specimens and also look at the effects of this surgery in other taxa, for instance, non-arboreal relatives of the kinkajou as well as other arboreal taxa.

## 1. Introduction

Kinkajous (*Potos flavus*) are small, arboreal carnivores native to Central and South America [1]. Unlike most carnivorans, they are predominantly frugivorous [1,2], have a remarkably prehensile tail [3,4], and highly flexible feet [1,4,5] with myological adaptations to support these [4,6,7]. Because kinkajous have gained popularity as an “exotic” pet [8,9], they have become subjected to practices to make them easier to handle, such as declawing through “onychectomy”, the surgical removal of the nail with part, or all, of the distal phalanx [10,11,12,13]. Recently, we documented the substantial myological effects of onychectomy on exotic felids [14]. However, the effects of this surgery on the muscular anatomy of an arboreal species that uses its limbs so extensively for grasping are heretofore unknown. To that end, in this paper, we document the muscle anatomy and architecture of a kinkajou that was declawed early in life on all four limbs compared to the anatomy of intact specimens as a case study to better understand the myological effects of onychectomies on this arboreal taxon and compare these effects to those found in declawed felids.

### 1.1. Myological Effects of Onychectomies

Onychectomy, the surgery commonly referred to as “declawing” an animal, is performed on animals for a variety of reasons. In household cats, it is often done to prevent scratching of people and property [15]. Nondomestic animals, such as exotic pets or animals in pay-to-play situations, are declawed to make them easier to handle, more convenient to own, or to appear safer in public interactions or entertainment industries [13,16,17]. Owners who have done this to kinkajous have claimed that it makes their scratching less damaging to furniture and less dangerous for people [18], and that it makes them less aggressive in general [19], though there is no empirical evidence to support those claims [18,19].

Onychectomy itself involves the removal of the distal phalanx either in part or completely, typically on the forelimbs though it can also be done on the hindlimbs [10,11,12,13]. This approach is controversial as in cats it has been linked to unwanted behaviors like biting and house soiling and reduces the cat’s welfare by limiting its ability to fully express its normal behavior and, when done inadequately, leads to discomfort and lameness caused by bone fragments left after surgery [20,21,22]. For this reason, domestic cat declawing is banned in many countries and some US states [23,24,25,26], while some non-domestic and exotic animals are protected under additional laws [25]. However, declawing kinkajous remains legal throughout the US.

The muscles that allow for flexion and extension of the claws originate in the antebrachium and crus, hereafter referred to as the forearm and leg respectively. In our previous research, we showed that the mass and physiological cross-sectional area (PCSA) of the digital extensors and flexors are significantly different in declawed felids compared to intact members of the family and that these effects are more substantial in larger species [14]. In felids, these muscles, in combination with others, are most significantly used for locomotion (including digitigrade posture as well as high demands in activities such as jumping), grasping in the form of prey grappling, and claw protraction [27,28,29,30]. Although the muscles that flex and extend the wrist also originate in the forearm, these are not substantially affected by onychectomy in felids, strongly suggesting that the differences in the anatomy of these muscles are related to their specific function in the digits [14].

While we have an understanding of how onychectomy affects the forearm muscle architecture in large and small felids, how this anatomy is affected in a grasping arboreal species is still unknown.

### 1.2. The Kinkajou

The kinkajou [1.4–4.5 kg; 1] is among eleven species in six genera of Procyonidae, the family that includes the raccoons (*Procyon*), olingos (*Bassaricyon),* ringtails (*Bassariscus*), and two genera of coatis (*Nasua* & *Nasuella*). Based on ecological and morphological characteristics, the procyonid genera have historically been split into two clades, with the more terrestrial *Nasua*, *Nasuella*, *Procyon*, and *Bassariscus* in one clade and the arboreal *Potos* and *Bassaricyon* in the other [31,32]. However, molecular phylogenetics suggests that these groupings represent some degree of convergence; although *Bassariscus* and *Procyon* appear to be sister taxa, *Nasua* and *Nasuella* are genetically most similar to the arboreal *Bassaricyon*, and their morphological similarity to *Potos*, which is genetically isolated from the other two clades of the family, appears to be homoplastic [33].

Arboreality, along with its extreme frugivory, are hallmarks of the taxon. Kinkajous spend 50–65% of their day traversing the forest, and are almost never on the forest floor, though they do make use of various levels of the canopy [1]. Considered to be important seed dispersers [34], their diet consists of an estimated 90–99% fruit, with the remaining 1–10% being a mix of flowers, leaves, nectar, and very rarely, insects [1,2].

Kinkajous are plantigrade [4] and move with an almost primate-like gait with a diagonal sequence [35] in a relatively crouched position, achieved not through limb shortening but through greater joint flexion [4]. However, their most striking locomotive adaptation is their highly dexterous prehensile tail which allows them to suspend themselves from branches and traverse gaps between trees similar to the locomotion pattern seen in spider monkeys (*Ateles*). The binturong (*Arctictis bintourong*) is the only other carnivoran that has a prehensile tail [3], but its tail is substantially less dexterous than that of the kinkajou.

Kinkajous also possess highly flexible ankles that allow for 180 degrees of rotation [1,4,5]. This increased mobility allows for headfirst descent [4,36] as well as upside-down suspension during foraging [36,37]. Furthermore, kinkajou hands are highly dexterous. However, like most carnivorans, their thumb is poorly differentiated from their other fingers and thus they grasp by folding all of their fingers inward, converging at the center of the palm, a motion similar to “pseudo-opposition” in primates [4].

### 1.3. Kinkajous as Exotic Pets

Unfortunately, kinkajous are relatively common as exotic pets. They are listed as a species of least concern and thus their international trade is relatively unregulated [38]. Although their ownership is restricted by some national and state laws, most often designed around the safety of humans [39], kinkajous have been estimated to be among the top five internationally traded species with an estimated 1599 individuals traded from 2006 to 2012 across the globe [40]. There are many sources on the internet for purchasing kinkajous, as kinkajous are currently legal and readily available in 35 US states [41]. While some sources advocate for them as excellent pets for the right type of person [41,42], there are concerns about animal welfare, environmental protection, public health, and pet and human safety [8,39,43]. Up to 90% are rehomed due to unexpected behavior, such as biting and clawing, as well as challenges surrounding upkeep, such as social, dietary, and grooming requirements [44]. Unfortunately, these issues lead to not just rehoming, but also declawing. However, even without their claws, kinkajous have powerful bites and are still considered dangerous [19,45].

### 1.4. A Case Study: The Myological Effects of Onychectomy in a Kinkajou

In this study, we document the muscle architecture of a declawed female kinkajou (AHR # 211061) that was born on 25 March 1988, and subsequently rehomed to an animal rescue facility on 24 March 1993. Prior to being rehomed, she was declawed on all of her front- and hindlimb digits. Because of severely declining health, she was euthanized on 2nd August 2023, and subsequently donated for this research.

During her life at the sanctuary, she was housed in a mixed indoor–outdoor enclosure with ample space and naturalistic structures on which to move and climb, as well as specific enrichment the facility uses for all of its kinkajous (e.g., climbing and foraging structures), though this was modified to accommodate her being declawed; her enrichment required less dexterity (e.g., food fed in containers had to be easier to open than for other kinkajous) and she was only given enrichment that did not require her to be fully suspended. By the end of her life, she had arthritis, especially in her manus—likely caused or exacerbated by her onychectomy—and had difficulty grasping. With that said, moderate arthritis (e.g., lipping) was evident in most of her joints, as is common for most animals at her life stage. More than a decade prior to her euthanasia, and prior to her enclosure being modified to accommodate her apparent reduced dexterity, the fourth digit on her right hand was amputated at the metacarpophalangeal joint due to complications from getting it caught in something that became a ligature. Due to her age, declaw-related pathology, and unrelated medical complications, she was receiving Acetylcysteine, Gabapentin, Gas-X, Guaifenesin, Metoclopramide, Miralax, and Prednisolone at the time of her death.

In this study, the anatomy of this specimen is compared to that of two female kinkajous with intact claws.

## 2. Hypotheses

Although the focal animal was geriatric and had arthritic pathology likely (but not definitively) related to its onychectomy, it is informative to examine myological differences between this specimen and intact kinkajou specimens—especially in comparison to our recent findings on the myological differences between clawed and declawed felids [14]. To this end, we test the following hypotheses:

**H1.**  *Based on our findings on clawed versus declawed felids [14], onychectomy will have an effect on the architecture of some, but not all, of the forearm muscles in the kinkajou.*

**H1a.**  *The forearm muscles that are associated with grasping and digital extension will have lower muscle mass and physiological cross-sectional area (PCSA; relative to body mass), in the declawed kinkajou as compared to the intact ones.*

**H1b.**  *The muscles not associated with grasping or digital extension will have equivalent relative muscle mass and PCSA, in the declawed kinkajou as compared to the intact ones. Alternatively, the muscles may show an increase in muscle mass and PCSA (relative to body mass), in the declawed kinkajou as compared to the intact ones, to compensate for the reduction of other forearm muscles. While this was not seen in felids [14], it may be true in kinkajous due to differences in digital use and locomotion [1,3,4,6].*

**H1c.**  *Relative fascicle lengths (FL) will not differ in the forearm between the declawed kinkajou and the intact ones.*

**H2.**  *Onychectomy will have a similar effect on the architecture of the forearm muscles of the declawed kinkajou as it does in declawed felids [14]. Specifically, the declawed kinkajou’s forearm muscles that are reduced, relative to body mass, in PCSA and mass are not only within the same functional category as those in felids but also the same individual muscles.*

**H3.**  *Based on our findings on the forearm muscle architecture in clawed versus declawed felids [14], onychectomy will have an effect on the architecture of some, but not all, of the leg muscles in the kinkajou.*

**H3a.**  *As predicted for the forearm muscles based on previous findings, the leg muscles that are associated with grasping and digital extension will have relatively lower muscle mass and PCSA in the declawed kinkajou as compared to the intact ones.*

**H3b.**  *Likewise, the leg muscles not associated with grasping or digital extension will have equivalent relative muscle mass and PCSA in the declawed kinkajou as compared to the intact ones. Alternatively, as with our prediction about the forearm muscles, the non-grasping leg muscles may show an increase in relative muscle mass and PCSA in the declawed kinkajou as compared to the intact ones, to compensate for the reduction of other leg muscles. While compensation was not seen in felid forearm muscles [14], it may occur in kinkajous as they rely heavily on finger movement in food procurement and arboreal grasping [4,6], and therefore may need to compensate.*

**H3c.**  *As predicted for the forearm muscles based on previous findings, relative leg FLs will not differ between the declawed kinkajou and the intact ones.*

**H4.**  *Onychectomy in the kinkajou will have different myological effects in the forearm and leg. Alternatively, because the taxon may heavily rely on both their fore- and hindlimbs so extensively in arboreal locomotion [1,3,4,6], the architecture of the forearm and leg muscles may be equally affected by onychectomy in kinkajous.*

**H4a.**  *Because of their use in food foraging and other manual grasping activities [4,6], activities curtailed in the declawed kinkajou, the relative muscles masses and PCSAs of the declawed kinkajou as compared to the intact ones will be more decreased in the muscles of the forearm than those of the leg.*

**H4b.**  *Alternatively, the kinkajou’s reliance on their forelimbs may mean that the leg muscles may be more affected by onychectomy because the declawed animal may have needed to maintain manual grasping abilities despite its alteration.*

## 3. Materials and Methods

### 3.1. Sample

We dissected the forearm and leg muscles of three captive female kinkajous, one declawed on all four limbs and two anatomically intact. Bilaterally dissected limb elements were averaged for each individual prior to analysis. The declawed specimen was declawed early in its life and many years prior to its death. All animals were humanely euthanized for reasons unrelated to this study.

**Figure 1:**
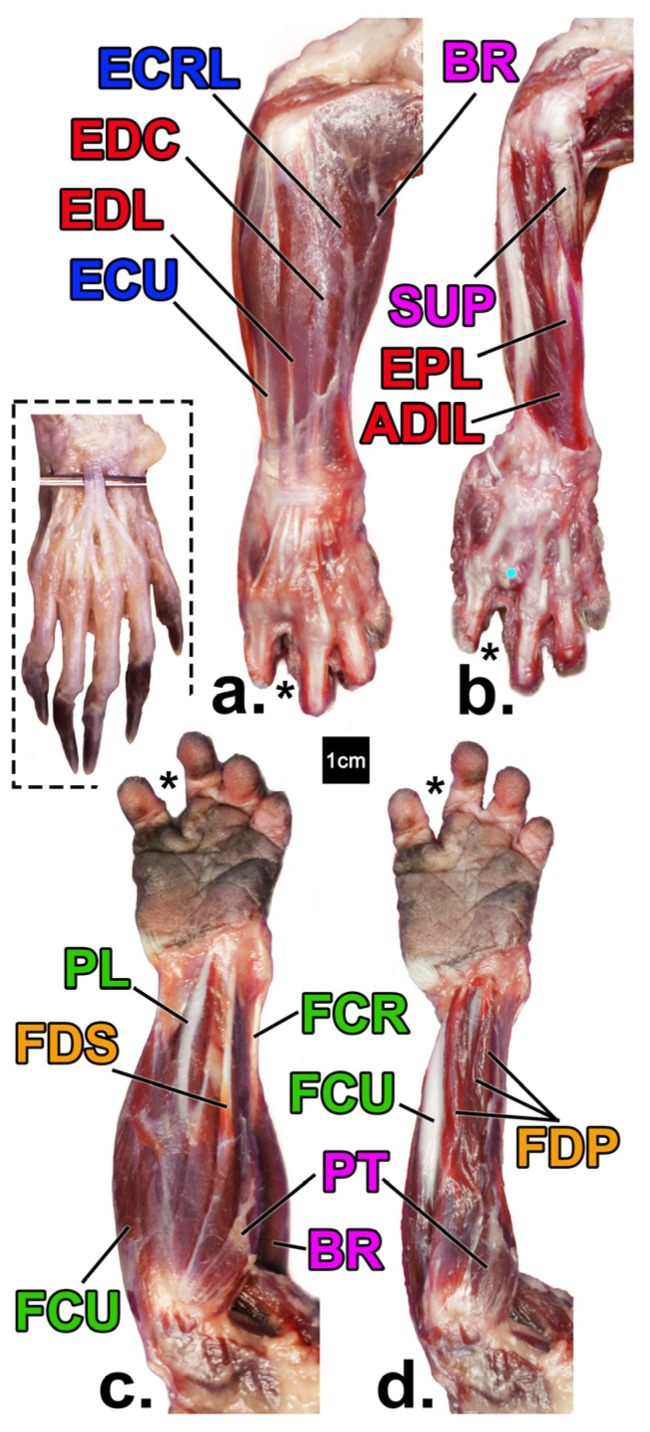
Forearm muscles of the declawed *Potos flavus* that is the focus of this paper (**a**–**d**) in comparison to that of an intact specimen (inset; from [47] courtesy of B. Townsend). Superficial (**a**) and deep (**b**) views of the posterior (extensor) compartments, and superficial (**c**) and deep (**d**) views anterior (flexor) compartments, respectively. Yellow = digital flexors; green = wrist flexors; red = digital extensors; blue = wrist extensors; pink = pronators/supinators/other. See Table 1 for muscle name abbreviations. ECRB is not pictured due to its position deep to BR. The asterisk denotes the position of where the amputated fourth digit would be with the cyan dot marking the head of metacarpal IV.

**Figure 2:**
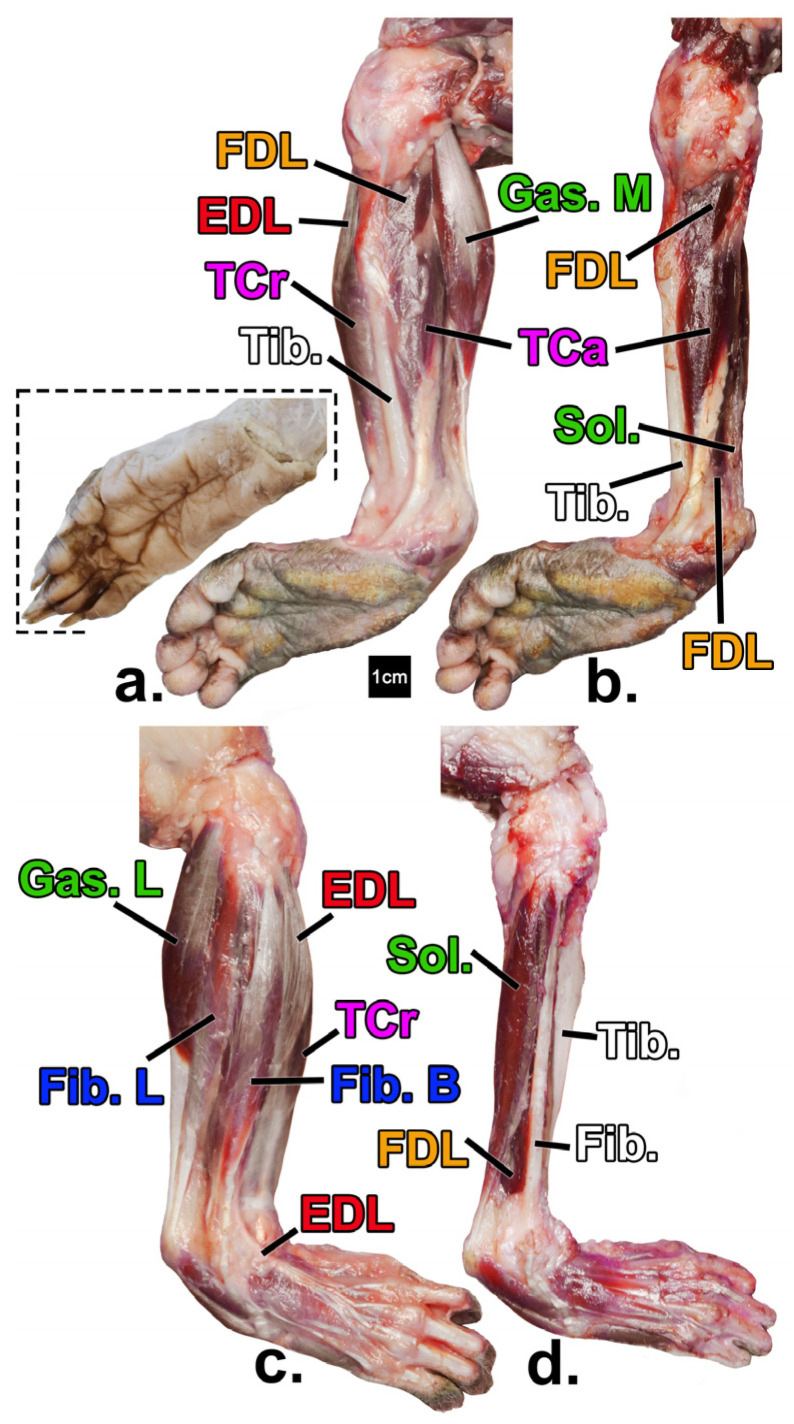
Leg muscles of the declawed *Potos flavus* that is the focus of this paper (**a**–**d**) in comparison to that of an intact specimen (inset; from [48] courtesy of H. Smith). Superficial (**a**) and deep (**b**) views of the medial compartments, and superficial (**c**) and deep (**d**) views of the lateral compartments, respectively. Yellow = digital flexors; green= non-digital plantar flexors; red = digital extensors; blue = evertors; pink = invertors; white = bone. See Table 1 for muscle name abbreviations.

Muscles of the forearm and leg were studied both individually and grouped by function (Table 1, Figure 1 and Figure 2). Nomenclature follows on Nomina Anatomica Veterinaria [46] when appropriate.

### 3.2. Data Collection

The three specimens were frozen fresh with the skin on and showed no evidence of freezer burn. Freezing and thawing in this manner has been shown to have no statistical effect on muscle architecture compared to fresh specimens that have never been frozen [49] and has been the predominant state of specimens that we have included in previous studies [14,50,51,52,53]. Following our previous research methodology in declawed felid forearms [14], all specimens were skinned, sharp dissected from the superficial to deep compartments, and muscles removed from origin to insertion, with tendons crossing over the carpus or tarsus being severed at the distal radius or tibia, respectively. For each muscle, linear measurements were recorded using digital calipers [14,54] and mass (to the nearest 0.00001 g) and density were recorded using a Mettler Toledo density scale MS105 XPR-S XSR-S 0.1 mg, 1 mg density kit to the nearest 0.001 g/cm^3^.

While body mass was known for the focal declawed specimen, the last known body mass for the comparative specimens was unknown. As these specimens appeared slightly smaller than average kinkajous, their body masses were estimated based on their femoral lengths relative to that of the declawed specimen using the following equation:

*BM_I_* = *F_I_*^3^ × (*BM_D_*/*F_D_*^3^)

where *BM* is the body mass of the intact (*I*) and declawed (*D*) kinkajous (estimated and known respectively), and *F* is their respective femoral lengths, which are cubed to account for the scaling difference between their linearity and the volume-related mass.

To separate the fascicles for measurement, chemical dissection was performed following Martens et al. [14], Herrel et al. [55], and Boettcher et al. [56]. In short, after collecting mass, density, and linear measurements of each muscle, it is placed in 25–35% nitric acid (smaller muscles disarticulated at the lower end of this range) at room temperature to dissolve the connective tissues until the muscle fascicles are easily manually separated. Depending on acid concentration, muscle size, and connective tissue content, this takes twelve to forty-eight hours for each muscle, after which the acid is replaced with a 50% glycerin solution to stabilize and cease digestion. Fascicles of each individual muscle are then gently physically separated from one another, photographed with a scale bar, and a subsample of ideally 10–40 (depending on muscle size) are measured using ImageJ 1.53 k.

### 3.3. Architectural Variables Studied

This sample is used to establish the average fascicle length (FL) of each muscle from which, along with its mass, the physiological cross-sectional area (PCSA) is calculated following Schumacher [57]:

*q* = *m*/*lp*

where *q* is PCSA (cm^2^), *m* is muscle mass (g), *l* is mean fiber length (cm), and *p* is muscle density (g/cm^3^). For the few muscles for which a direct density measurement was not available (e.g., muscles that were too small or too large to accurately measure in the system we used), a density proxy was calculated by averaging densities of muscles by functional group for intact and declawed animals separately and then assigning that value as the proxy for muscles within that functional group.

Following our previous approaches [14,56,58,59], target muscles of interest (e.g., those specifically most associated with the distal phalanges) were analyzed individually, and all muscles were also analyzed in functional groups (see Table 1). Total muscle masses and PCSAs were calculated by summing the muscle mass or PCSA of all muscles in each functional group. Weighted average FL was calculated for each functional group following Leischner et al. [59]:

$$\frac{\sum_{n=1}^{i}{FL}_{n}\times {MM}_{n}}{\sum_{n=1}^{i}{MM}_{n}}$$

where *FL_n_* and *MM_n_* are the muscle mass and *FL* of each muscle *n* in that group.

Also following Martens et al. [14] and Leischner et al. [59], prior to analysis, variables were linearized by taking square and cubic roots of the area (PCSA) and volume-related (mass) variables. Each was then divided by the cubic root of the relativized body mass (kg) of the respective specimen to reduce the influence of overall body size when comparing the specimens relative to each other. When left and right fore- or hindlimbs of a specimen were available, they were averaged to create one comparative value for each specimen. Proportional differences between intact and declawed kinkajous were also calculated, with the declawed value divided by the intact value, to better represent the results.

To compare kinkajou data to that of the felids, ordinary least squares regressions were run from the felid data from Martens et al. [14] and predictions of muscle masses, PCSA, and FL were made for an average theoretical intact and declawed felid of the sample. Proportional differences of these declawed and intact felid values were calculated in the same way as for the kinkajous. Comparisons for all available muscles and variables were made and reported, though statistically significant felid values are highlighted (Table 2).

All statistical analyses were performed in JMP Pro 17 (SAS).

## 4. Results

### 4.1. Forearm Muscle Differences (Muscle Mass, Physiological Cross-Sectional Area, and Fascicle Length)

In many cases, the declawed kinkajou had relatively larger muscle masses of forearm muscles when compared to those of the intact individuals (Table 2). This was true for all functional groups but one, which ranged from 1.04 to 1.21 times (for the wrist extensors and flexors, respectively) relatively larger in the declawed kinkajou. The one exception was the digital extensor group, for which the declawed kinkajou was relatively smaller—93% of the relative mass of the intact specimen average. A similar trend followed for most of the individual muscles with the declawed specimen’s muscles ranging from 1.01 to 2.09 times the relative mass (for FDP and PL respectively) of the intact specimens except the carpal flexors (FCR, FCU), and PQ, for which the declawed specimen’s muscles were 69–90% the size of those of the intact specimens. Surprisingly, wrist flexors as a functional group remained relatively larger in the declawed kinkajou, even with a relatively smaller FCU and FCR because the specimen’s PL (more than double that of the intact specimens) more than made up for the carpal flexor relative mass deficit.

Interestingly, although mass and PCSA usually have similar trends [14,52,53,60], the relative forearm muscle PCSA trends were notably different: The digital functional groups and the total flexors had a smaller relative PCSA in the declawed kinkajou, by a factor of 0.93 (digital flexors) to 0.98 (digital extensors; Table 2). The other functional groups ranged from 1.00 (total flexors) to 1.18 (wrist extensors)—relatively larger in the declawed kinkajou. The individual muscles ranged from relatively smaller by a factor of 0.87 (ECRB) to 2.71 times relatively larger (PL) for the declawed specimen. Interestingly, the relatively smallest functional group in the declawed kinkajou, the digital flexors, was driven by FDS and FDP, 89% and 91%, respectively, the strength of those of the intact specimen. This caused the digital flexors to be 93% of the size of those in the intact kinkajous in the declawed specimen. It is also worth noting that ECRL and ECRB are trending in opposite directions, with ECRL being 1.45 times relatively larger and ECRB relatively smaller by a factor of 0.87 in the declawed kinkajou.

Average fascicle lengths (FL) by functional group tended to be relatively longer in the declawed kinkajou, up to 1.35 (wrist extensors) times those of the intact specimens, other than in the wrist flexor and digital extensor groups, for which the declawed kinkajou’s FLs are relatively shorter—by a factor of 0.87 and 0.89, respectively (Table 2). In the individual muscles, FLs, ranged from relatively shorter by a factor of 0.43 (PQ) to 2.00 times relatively longer (ECRB) in the declawed specimen.

### 4.2. Comparison to Felids

**Figure 3:**
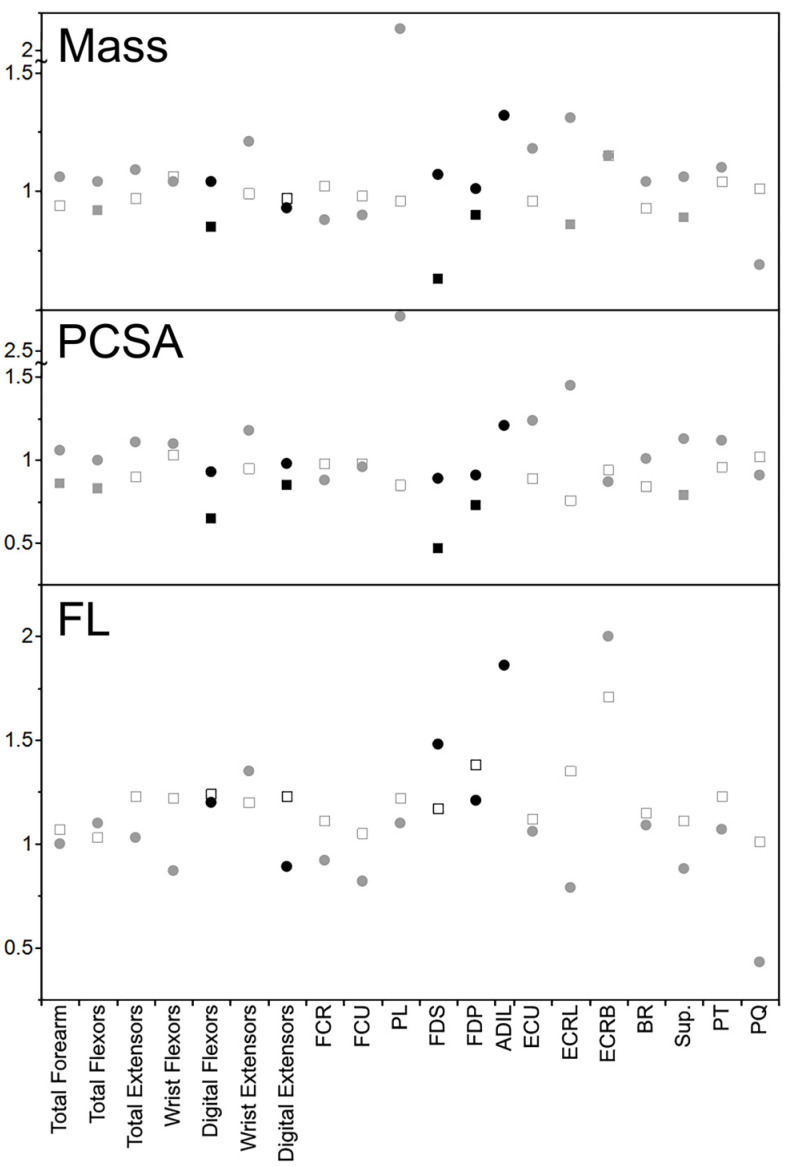
Relative declawed forearm muscle mass, PCSA, and FL of the kinkajou compared to that of felids. The circles represent kinkajou values and the squares felid values. Black shapes represent muscles/functional groups that most directly relate to digital function. Filled squares are statistically significantly different in declawed versus clawed felids. Likely due to sample size, no kinkajou values were statistically significantly different between the declawed and clawed specimens.

In the kinkajou forearm, the muscle masses of the declawed specimen range from relatively smaller by a factor of 0.93 (digital extensors) to 1.21 times relatively larger (wrist extensors; Table 2). All functional groups other than digital extensors were relatively larger in the declawed kinkajou. This is quite dissimilar to what was found in the felids from Martens et al. [14], where the muscle masses of the declawed specimen ranged from relatively smaller by a factor of 0.85 (digital flexors) to 1.06 times relatively larger (wrist flexors), with only one functional group being relatively larger in the declawed felids (Figure 3). The dissimilar trend continues in the individual muscles, with the muscle mass ranging from relatively smaller by a factor of 0.69 (PQ) to 2.09 (PL) times relatively larger in the declawed kinkajous but relatively smaller by a factor of 0.63 (FDS) to 1.15 times relatively larger (ECRB) in the declawed cats. Even more interestingly, the muscles that had significantly relatively smaller muscles masses in the felids—total flexors, digital flexors, FDS, FDP, ECRL, and Sup.—were relatively larger in the declawed kinkajou. And PL, the muscle relatively larger by the most in the declawed kinkajou (2.09 times), is relatively slightly smaller by a factor of 0.96 in the declawed felids.

In PCSAs, the trends between the declawed and intact kinkajous and felids are more similar. In the kinkajou forearm functional groups, the PCSAs ranged from relatively smaller by a factor of 0.93 (digital flexors) to 1.18 times relatively larger (wrist extensors) in the declawed kinkajou (Table 2). Digital extensors and digital flexors both had lower relative PCSAs in the declawed kinkajou, and other than that, the functional groups were relatively equal or larger in the declawed kinkajou. While the PCSAs of the wrist flexor functional group of the declawed felids were slightly relatively larger (1.03 times), all the other functional groups had relatively smaller PCSAs in the declawed felids, the smallest (by a factor of 0.65) found, as was expected based on the nature of onychectomy, in the digital flexors.

In the kinkajou individual forearm muscles, the PCSAs vary from relatively smaller by a factor of 0.87 (ECRB) to 2.71 times relatively larger (PL) in the declawed kinkajou. In the felids, all individual muscles were relatively smaller in declawed animals by up to a factor of 0.47 in the deep digital flexor (FDS), which attaches to the distal phalanx in intact specimens, and only the deepest and relatively small *pronator quadratus* (PQ) was slightly larger (1.02 times) in the declawed felids. While PL trends extremely differently in the kinkajou versus felid samples, some of the other muscle PCSAs trend in the opposite direction as well. Namely, the relative PCSAs of the declawed compared to intact kinkajous were notably different than those of felids in their ECU, ECRL, BR, Sup., PT, and PQ. Additionally, the muscles that had relatively significantly lower PCSAs in the declawed felids by the most, FDS and FDP, were also relatively smaller in the declawed kinkajous.

For the FLs, the trends are somewhat similar again. In the kinkajous, the FLs by forearm functional group ranged from relatively shorter by a factor of 0.87 (wrist flexors) to 1.35 (wrist extensors) times relatively longer in the declawed specimen (Table 1). For felids, the FLs by forearm functional group were relatively longer in the declawed felids for all groups, ranging from 1.03 (total flexors) to 1.24 times relatively longer (digital flexors). While some of the individual kinkajou muscle FLs are relatively shorter (by a factor of up to 0.43 in PQ) and most are relatively longer (up to 2.00 times relatively longer in ECRB), the individual FLs of the declawed felids were all relatively longer ranging from 1.01 (PQ) to 1.71 (ECRB) times relatively longer. However, none of these differences were statistically significant for the FLs in the intact and declawed felids [14].

### 4.3. Leg Muscle Differences (Muscle Mass, Physiological Cross-Sectional Area, and Fascicle Length)

**Figure 4:**
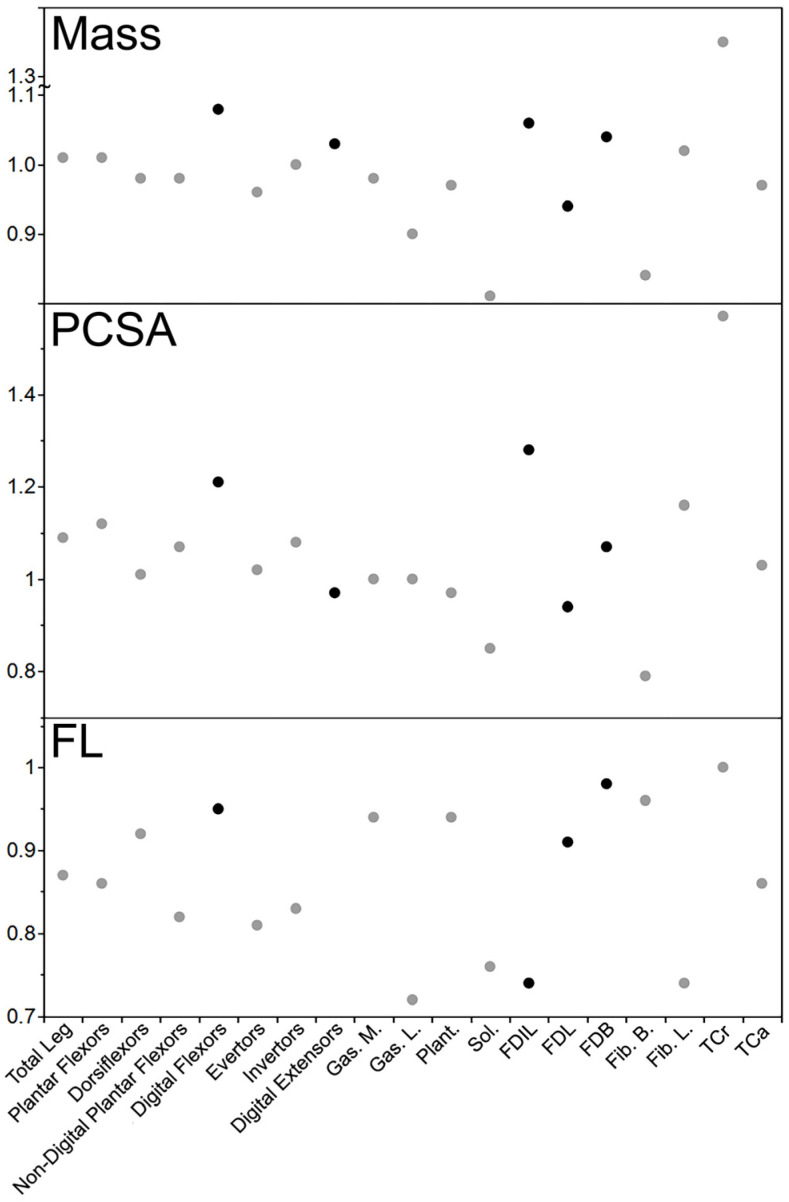
Relative declawed leg muscle mass, PCSA, and FL of the kinkajou. Black circles represent muscles/functional groups that most directly relate to digital function. As with the forearm comparisons, likely due to sample size, no kinkajou values were statistically significantly different between the declawed and clawed specimens.

In the leg, the muscle masses of the declawed kinkajou were quite similar to those of the intact kinkajous, ranging from relatively slightly smaller (by a factor of 0.96 for the evertors) to slightly relatively larger (1.08 times for the digital flexors; Table 3 and Figure 4). Contrary to the hypothesis, the digital muscles of the declawed kinkajou were relatively larger by the most, with the digital flexors 1.08 times relatively larger and the digital extensors 1.03 times relatively larger. The individual muscles follow this trend, from relatively smaller (by a factor of 0.81 for Sol.) to relatively larger (by 1.35 for TCa) in the declawed kinkajou. The muscles that were relatively smaller by the most in the kinkajou (Sol. = 0.81 and Fib. B = 0.84) drive the trend seen in the non-digital plantar flexors and evertors. Interestingly, certain functional synergist pairs trend in opposite directions with the declawed specimen’s FDL being relatively smaller by a factor of 0.94, while FDB was 1.04 times relatively larger, and Fib. B was relatively smaller by a factor of 0.84, but Fib. L was 1.02 times relatively larger.

In terms of PCSAs of functional groups, the declawed kinkajou had a relatively larger PCSA for all functional groups, ranging from 1.01 (dorsiflexors) to 1.21 (digital flexors) times the relative area (Table 3). In the individual muscles, the trend is more varied, from relatively smaller by a factor of 0.79 (Fib. B) to 1.57 times relatively larger (TCa) in the declawed individual. As with their masses, the functional synergists FDL and FDB (respectively 0.94 and 1.07 times those of the intact specimens) and Fib. B and Fib. L (0.79 and 1.16 respectively) tended in opposite directions. 

The FLs of all functional groups and individual muscles were relatively shorter in the declawed kinkajou, except for one group and one individual muscle (Table 3). In the functional groups, they ranged from relatively shorter by a factor of 0.81 (evertors) to 0.95 (digital flexors) in the declawed kinkajou, other than digital extensors which were 1.17 times relatively longer. The individual muscles ranged from relatively shorter by a factor of 0.72 (Gas. L) to 0.98 (FDB) in the declawed kinkajou, other than TCa in which the FLs were relatively the same size in both the intact and declawed kinkajous.

### 4.4. Forearm Compared to Leg

In the kinkajou forearm, the muscle masses of the declawed specimen ranged from relatively smaller by a factor of 0.93 (digital extensors) to 1.21 times relatively larger (wrist extensors; Table 2). All functional groups other than digital extensors were relatively larger in the declawed kinkajou. In the leg, the muscle masses of the declawed kinkajou were relatively quite similar to those of the intact kinkajous, ranging from relatively smaller by a factor of 0.96 (evertors) to 1.08 times relatively larger (digital flexors) in the declawed kinkajou (Table 3). The larger range of forearm muscle masses continues in the individual muscles, with the muscle mass ranging from relatively smaller by a factor of 0.69 (PQ) to 2.09 (PL) times relatively larger in the declawed kinkajou’s forearms, but a narrower range of 0.81 to 1.35 times (Sol. and TCa, respectively) in the declawed kinkajou’s legs.

This larger range in the forelimb continues in the PCSA. In the kinkajou forearm, the PCSAs of the functional groups ranged from 0.93 to 1.18 times relatively (digital flexors and wrist extensors, respectively; Table 2) and 1.01 to 1.21 times relatively larger (dorsiflexors and digital flexors, respectively; Table 3) in the leg—all relatively higher in the declawed specimen, but spanning a narrower range of variation. Interestingly, where the digital functional groups seemed to be relatively smallest by the most in the forearm, only the digital extensors were relatively smaller in the declawed kinkajou leg at all, and the digital flexors were relatively larger in the declawed kinkajou leg by the most (Table 2 and Table 3).

For the FLs, the trends were quite different in the forearm and leg: The FLs by forearm functional group ranged from relatively shorter by a factor of 0.87 (wrist flexors) to 1.35 (wrist extensors) times relatively longer in the declawed kinkajou (Table 2) but this range was relatively much narrower in the leg—0.81 to 1.17 times relatively (evertors and digital extensors, respectively; Table 3). In the forearm of the declawed specimen, all but two functional groups had relatively longer or equal FLs, but in the leg, this was almost reversed with all but one functional group having relatively shorter FLs. For the individual muscles, FLs ranged from relatively shorter by a factor of 0.43 to 2.00 times relatively (PQ and ECRB, respectively) in the forearm, and again across a narrower range of 0.72 to 1.00 (Gas. L and TCa, respectively) in the leg.

## 5. Discussion

We found support for some of our hypotheses and can reject others: While the architecture of some of the muscles of the declawed kinkajou was substantially different from that of the intact specimens (supporting H1), and as was seen in felids, the digital muscles have reduced PCSA (supporting H2), unlike in the felids, the declawed kinkajou had relatively more massive forearm muscles than those of the intact specimens (contrary to H2) and (contrary to H3) the leg muscle architecture of the declawed kinkajou was not substantially different from that of the intact specimens. This substantial difference in the response of onychectomy in the forearm versus leg muscles supports H4—specifically H4a, suggesting that the kinkajou’s differential limb usage means that this surgery has a much more significant effect on its forelimb.

### 5.1. Forearm Muscle Differences

It is noteworthy that, aside from the digital extensors, all forelimb functional groups have relatively larger muscle masses in the declawed kinkajou, only partially supporting H1a. However, other than wrist extensors, they are not larger by much (showing support for H1b). It is noteworthy that the digital extensor group is the only functional group that is relatively smaller in the declawed kinkajou and the functional group that is relatively larger by the most in the declawed kinkajou is the wrist extensor group. These two muscle groups both extend, but across different joints; perhaps this reflects compensation (H1b). That is, if the digital extensors are most affected by onychectomy as the extensor aponeurosis onto which the digital muscles insert is disrupted, perhaps the wrist extensors somehow compensate for the functional diminution of the digital extensors. However, it is surprising then that the digital flexor group (which includes the FDS that normally inserts on the distal phalanx) is not smaller in the declawed kinkajou. Why the digital extensors but not the digital flexors would be substantially affected in the kinkajous, especially since this is the opposite pattern of what was seen in the felids, is hard to explain functionally.

The most dramatic finding is that though both carpal flexors (FCR and FCU) are relatively smaller in the declawed kinkajou, its PL more than makes up for this. As in most mammals that have it, the *palmaris longus* inserts into the connective tissue between the palmar dermis and the palmar muscles and extrinsic digital flexor tendons. In most arboreal mammals (e.g., primates and rodents), including intact kinkajous, it is among the smallest of the flexor compartment muscles—generally substantially smaller than its synergist carpal flexors. Why the dedicated carpal flexors, which do not attach to any of the phalanges, no less the distal ones that are removed in onychectomy, would be so dramatically reduced in the declawed kinkajou is unclear, as is the reason that this movement would be compensated for by an increase in the usually small PL. It is possible that this is a peculiarity of the declawed individual specimen in particular, but if this is an example of one muscle compensating for losses in other muscles, it is not compensatory in any way that seems predictable. Clearly, this anatomy should be examined in other clawed and declawed specimens to see if these carpal flexor differences are generalizable.

Although they were more massive, the physiological cross-sectional areas (PCSA) of the forearm digital flexors and extensors are relatively smaller in the declawed kinkajou, partially supporting H1a. This finding aligns well with the idea that declawing reduces digital function: Removing the insertion of digital muscles must affect the behavior capable with the amputated digits, thus reducing the need for strength in the digit-specific muscles, as reflected in their reduced PCSAs. Interestingly, it appears that the weaker digital muscles are accompanied by the stronger wrist muscles. This supports the compensation theory (H1b); perhaps the manual behaviors (especially postural ones) that are normally enabled by digital manipulation (made difficult with the removal of the distal phalanx) are accomplished in the declawed specimen through use of its wrist instead, as is reflected by its stronger wrist muscles.

What is even harder to explain is our finding that some muscle groups and individual muscles trend in opposite directions between their masses and PCSAs; specifically, the declawed kinkajou generally had relatively larger forearm muscle masses, but relatively lower PCSAs. As these both reflect force adaptations generally scale in the same direction [14,50,52,53,60,61], it is unclear why a relatively larger muscle mass would not be accompanied by a relatively larger PCSA. Rasch [62] suggested that while there is a relationship between hypertrophy and increased strength, it is unlikely that a direct correlation exists. Furthermore, while much of an increase in muscle strength can be explained by hypertrophy, other, more poorly understood factors are also at play [63]. Ahtiainen et al. [64] showed that while resistance training can increase both muscle size and strength, it does not do so at the same rate. Therefore, while PCSA and muscle mass are often closely related to one another, these factors must be thought of more distantly from one another in order to understand what is occurring in the declawed kinkajou, especially in its digital flexors.

As PCSA is a product of mass and FL (along with muscle density), the discordance in the PCSA and mass trends in the declawed and intact specimens is partially explained by the differences in average FLs between the specimens. Contrary to what was generally seen in the felids and predicted in H1c, there were substantial differences in relative FLs between the declawed and intact specimens, and although average FLs tended to be relatively longer in the declawed specimen (explaining its relatively lower PCSA), there seemed to be no discernable functional pattern explaining the FL differences. In general, longer fascicles allow greater relative excursion [50,60,65] or speed [66,67], but the FL differences between the clawed and declawed kinkajous (Table 2) do not seem to follow a predictable trend.

### 5.2. Comparison to Felids

Other than for the digital extensors, the declawed kinkajou had relatively larger forearm muscle masses than did the intact kinkajous, a significant trend not seen at all in the felids, rejecting H2. This is perhaps because of different forearm functions in felids and kinkajous. In both, the forelimbs contribute to weight-bearing, but how they do so differs: Plantigrade kinkajous use their forelimbs primarily for arboreal grasping (often on vertical supports) while fewer felids are habitually arboreal and most generally use their digitigrade limbs for more horizontal quadrupedal locomotion [1,4,27,28,29,35,68]. While kinkajous use their grasping limbs more often for locomotion than do felids, felids use their forelimbs more extensively (and forcefully) for prey capture and subduction [68] and have evolved anatomical structures that allow effective claw retraction [69,70]. Thus, though the behavioral and functional differences between these taxa may explain why declawing apparently affected them differently, why the declawed kinkajou had a relatively greater forelimb muscle mass while the felids did not show this at all, remains unclear.

However, the kinkajous and felids did show similarities in terms of their forearm force capabilities as reflected by their muscle PCSAs: Both showed, as would be expected, relatively smaller PCSAs in their digital flexors (FDS and FDP) in the declawed specimens, driving changes in the digital flexor, total flexor, and overall forearm groupings, as well as relatively smaller digital extensors. Thus, while the muscle mass pattern was inexplicable, the PCSA findings were as expected and support H2. The similarities in the PCSAs of the digital flexors are likely explained by the fact that even though these muscles play different roles in kinkajous and cats—in kinkajous, they are more for grasping [35,71], and in felids, they are more for claw unsheathing [69,70]—they are still responsible for behaviors that apparently become absent or reduced after onychectomy. However, while the felids did not seem to compensate for the loss of digital force, it appears that kinkajous may do so by increasing force at the wrist. This may be because key felid digital activities (e.g., claw protraction) that are lost in declawing cannot be compensated for and without the most distal phalanx, there may be no possible compensation for digitigrade gait. Indeed, some declawed felids end up walking more plantigrade (i.e., with their wrists and ankles on the ground) or, in more extreme cases, even end up walking with their elbows on the ground—as was the case for a leopard (*Panthera pardus*) that was declawed prior to being in our care and subsequently anatomically studied. However, in the non-digitigrade kinkajous, compensation seems possible. Without the most distal phalanx, grasping, climbing, and walking likely become more challenging, but perhaps can be made easier by emphasizing more of the load of these activities on the wrist muscles. For example, perhaps without a distal phalanx, the wrist flexors play a greater role in locomotion and even aid in grasping food items. In this sense, it is not the onychectomy itself that directly dictates how an animal’s morphology responds, but whether this behavior is lost due to the onychectomy and whether this behavioral change can be morphologically compensated for by coopting other anatomy.

### 5.3. Leg Muscle Differences and Comparison of Onychectomy Effects in the Forearm and Leg

Although the declawed kinkajou had substantially different forearm muscle architecture, its leg muscles do not seem to have been significantly affected by its onychectomy, refuting H3. This is likely due to the fact that the leg is not used much for gripping and climbing, but more for walking and weight-bearing [35] and a lot of the grip that does occur in the hind end comes from the prehensile tail [1,4].

Overall, the leg muscles of the declawed and clawed kinkajous are much more similar than are their forearms, supporting H4a. While some of the muscle masses and PCSAs are slightly smaller in the declawed kinkajou, and some slightly larger, these differences are not very substantial. This is likely due to the reliance of the kinkajou on the forearm for grasping and climbing more than the leg [35]. Perhaps removing the distal phalanx does not affect plantigrade gait much, as the kinkajou is not walking on the distal phalanx but on the foot [4]. This may mean that the leg does not experience as much of a behavioral change as a result of declawing, neither in terms of grasping (since this isn’t a substantial leg behavior) or walking (since the digits are less emphasized in the plantigrade gait), and thus there is less of a morphological effect of onychectomy in the hindlimb than in the forelimb.

### 5.4. Limitations and Future Directions

The clearest limitation of this study is its sample size; although there are unfortunately many kinkajous in captivity, and even more unfortunately, many of these have undergone onychectomy, specimens of these long-lived animals—especially the declawed ones—are still rather rare to obtain for anatomical study. It is our hope that this study of this one declawed individual can serve as a preliminary analysis of the effects of this surgery on the anatomy of this taxon. However, analyses of other declawed specimens would help clarify several outstanding questions. Of course, additional specimens would allow us to confirm whether there are variable anatomical responses to this surgery. Additional specimens would also allow assessment of whether any of the apparently anomalous myological findings from this one declawed specimen were specific to her and potentially even completely unrelated to her onychectomy. For instance, perhaps her small carpal flexors and large palmaris longus were simply a peculiarity of this individual and had nothing to do with the surgery—something that seems potentially likely given that these muscles do not attach to the removed distal phalanx or any of the phalanges. If another declawed kinkajou fascinatingly showed this same wrist flexor pattern, then that would require more substantial consideration about the functional relationship between a digit surgery and wrist specialization—not just in overall magnitude of flexion abilities (something that makes sense given that the wrist is apparently compensating for damage to the grasping apparatus)—but why the palmar muscle would be emphasized at the apparent expense of the dedicated carpal flexors. Furthermore, additional specimens would allow better contextualization of which of the observed anatomy might be related to animal size (the focal declawed specimen was at the large body size range for the taxon and thus studying smaller declawed specimens and/or larger intact ones would be informative) and age—the focal specimen was arthritic—a state that, at least in part, appears to have been exacerbated by its onychectomy—but studying other younger declawed specimens and other older intact ones—especially ones with arthritis—would allow further extrapolation of the causal relationship leading to these anatomical signals.

Beyond expanding the intraspecific sample, it would be interesting to compare myological responses to limb damage in other prehensile tailed taxa relative to non-prehensile tailed analogues. For instance, while we have a rather detailed understanding of the effects of onychectomy in felid forearms [14], it would be valuable to compare these kinkajou findings to declawed and intact individuals of other procyonid species—e.g., the somewhat fossorial coati (*Nasua* spp.) or locomotor/manual generalist raccoons (*Procyon* spp.)—neither of which have prehensile tails. It is possible that onychectomy may have a less substantial effect on them in general since they presumably rely less on grasping in their more terrestrial locomotion style, or perhaps their hindlimbs would be more greatly impacted than those of kinkajous as they do not have prehensile tails to compensate for digital damage. Likewise, it would be interesting to examine the effects of digital damage in monkeys with and without prehensile tails since these are even better functional analogues to kinkajous than are their closest phylogenetic relatives. Again, these taxa are relatively long-lived, and they are not commonly subjected to onychectomy, but if any specimens become available that have substantial digital damage, it would be interesting to examine whether their myology follows any of the patterns seen in this focal kinkajou.

## 6. Conclusions

Although our case study was based on an examination of only one declawed specimen compared to the anatomy of intact conspecifics, we found that the architecture of some of its muscles—especially those of its forearms—was substantially different than that of the intact specimens, and as was seen in felids, the digital muscles appear to have been weaker. However, unlike in the felids, the declawed kinkajou had more massive forearm muscles than those of the intact specimens. Contrary to what we found in the forearms, the leg muscle architecture of the declawed kinkajou was not substantially different than that of the intact specimens, suggesting that the kinkajou’s differential limb usage means that this surgery has a much more significant effect on its forelimb. Future analyses should confirm the extent to which these findings are generalizable beyond this individual specimen and also look at the effects of this surgery in other taxa—especially non-arboreal relatives of the kinkajou (e.g., the procyonid coati and raccoon) and other arboreal kinkajou-like taxa like monkeys, with and without prehensile tails—if specimens can be identified with extensive digital damage. This study affirms, as expected, that at least one arboreal grasping taxon with a prehensile tail is substantially myologically affected by onychectomy (especially in its forelimbs) and the effects of this surgery or other manual abnormalities should be considered for other taxa with similar adaptations in comparison to those with different locomotor and hand use patterns. 

## Figures and Tables

**Table 1:** Muscles Studied.

Limb Segment	Broad Functional Group	Functional Subgroup	Muscle Name	Muscle Abbreviation
Forearm	Flexors (Forearm)	Wrist Flexors	*Flexor carpi radialis*	FCR
			*Flexor carpi ulnaris*	FCU
			*Palmaris longus*	PL
		Digital Flexors	*Flexor digitorum superficialis*	FDS
			*Flexor digitorum profundus*	FDP
			*Abductor digiti I longus*	ADIL
	Extensors (Forearm)	Wrist Extensors	*Extensor carpi ulnaris*	ECU
			*Extensor carpi radialis longus*	ECRL
			*Extensor carpi radialis brevis*	ECRB
		Digital Extensors ^a^	^a^	^a^
	Other		*Brachioradialis*	BR
			*Supinator*	Sup.
			*Pronator teres*	PT
			*Pronator quadratus*	PQ
Leg	Plantar Flexors	Non-Digital Plantar Flexors	*Gastrocnemius medialis*	Gas. M
			*Gastrocnemius lateralis*	Gas. L
			*Plantaris*	Plant.
			*Soleus*	Sol.
		Digital Flexors	*Flexor digiti I longus*	FDIL
			*Flexor digitorum longus*	FDL
			*Flexor digitorum brevis*	FDB
		Evertors	*Fibularis brevis*	Fib. B
			*Fibularis longus*	Fib. L
		Invertors	*Tibialis caudalis*	TCa
	Dorsiflexors		*Tibialis cranialis*	TCr
		Digital Extensors ^a^	^a^	^a^

^a^ Digital extensors of the first manual digit (EDIL) and *extensor digitorum communis*, *lateralis*, and *longus* (EDC, EDLa, and EDLo, respectively) in the cranial and caudal limbs as seen in the individual in Figure 1 and Figure 2, as well as any other dedicated extensor muscle, vary bilaterally and between individuals—some only have common extensors while others have accessory muscles that send tendons to one or multiple digits (e.g., “ED III” which extends the middle finger)—and are therefore analyzed only at higher grouping levels.

**Table 2:** Proportion ^a^ (declawed/clawed) of relative forearm muscle mass, PCSA, and FL by functional groups and muscle by muscle of intact and declawed kinkajous and felids (for comparison).

	Relative MM		Relative PCSA		Relative FL	
	Kinkajous	Felids	Kinkajous	Felids	Kinkajous	Felids
Total Forearm	1.06	0.94	1.06	0.86 *	1.00	1.07
Total Flexors	1.04	0.92 *	1.00	0.83 *	1.10	1.03
Total Extensors	1.09	0.97	1.11	0.90	1.03	1.23
Wrist Flexors	1.04	1.06	1.10	1.03	0.87	1.22
Digital Flexors	1.04	0.85 *	0.93	0.65 *	1.20	1.24
Wrist Extensors	1.21	0.99	1.18	0.95	1.35	1.20
Digital Extensors	0.93	0.97	0.98	0.85 *	0.89	1.23
FCR	0.88	1.02	0.88	0.98	0.92	1.11
FCU	0.90	0.98	0.96	0.98	0.82	1.05
PL	2.09	0.96	2.71	0.85	1.10	1.22
FDS	1.07	0.63 *	0.89	0.47 *	1.48	1.17
FDP	1.01	0.90 *	0.91	0.73 *	1.21	1.38
ADIL	1.32	^b^	1.21	^b^	1.00	^b^
ECU	1.18	0.96	1.24	0.89	1.06	1.12
ECRL	1.31	0.86 *	1.45	0.76	0.79	1.35
ECRB	1.15	1.15	0.87	0.94	2.00	1.71
BR	1.04	0.93	1.01	0.84	1.09	1.15
Sup.	1.06	0.89 *	1.13	0.79 *	0.88	1.11
PT	1.10	1.04	1.12	0.96	1.07	1.23
PQ	0.69	1.01	0.91	1.02	0.43	1.01

^a^ See Supplemental Table S1 for the size of all of these variables relative to body mass. ^b^ Felids do not have an *abductor digiti I longus* * Indicates statistical significance at *p* = 0.05 in the felids.

**Table 3:** Proportion ^a^ (declawed/clawed) of relative leg muscle mass, PCSA, and FL by functional groups and muscle by muscle of intact and declawed kinkajous.

	Relative MM	Relative PCSA	Relative FL
Total Leg	1.01	1.09	0.87
Plantar Flexors	1.01	1.12	0.86
Dorsiflexors	0.98	1.01	0.92
Non-Digital Plantar Flexors	0.98	1.07	0.82
Digital Flexors	1.08	1.21	0.95
Evertor	0.96	1.02	0.81
Invertors	1.00	1.08	0.83
Digital Extensors	1.03	0.97	1.17
Gas. M	0.98	1.00	0.94
Gas. L	0.90	1.00	0.72
Plant.	0.97	0.97	0.94
Sol.	0.81	0.85	0.76
FDIL	1.06	1.28	0.74
FDL	0.94	0.94	0.91
FDB	1.04	1.07	0.98
Fib. B	0.84	0.79	0.96
Fib. L	1.02	1.16	0.74
TCa	1.35	1.57	1.00
TCr	0.97	1.03	0.86

^a^ See Supplemental Table S2 for the gross size of all of these variables relative to body mass.

## Data Availability

Raw data and osteological remains of all specimens used in this study are available upon request from the corresponding author.

## Supplementary Materials

The following supporting information can be downloaded at: https://www.mdpi.com/article/10.3390/ani14192774/s1, Table S1: Relative forearm muscle mass, PCSA, and FL for functional groups and individual muscles for the declawed and intact kinkajous; Table S2: Relative leg muscle mass, PCSA and FL for functional groups and individual muscles for the declawed and intact kinkajous.
