# Supplementary material for: The Effects of Onychectomy (Declawing) on Forearm and Leg Myology in a Kinkajou (Potos flavus)
Source: Animals (Basel). 2024 Sep 26;14(19):2774. doi: 10.3390/ani14192774 (PMC11475305; doi:10.3390/ani14192774)
Supplement: Supplementary file 1 [file animals-14-02774-s001.zip › animals-3192589-supplementary.pdf]

Supplemental Table S1. Relative forearm muscle mass, PCSA and FL for functional groups and individual muscles for the declawed and intact kinkajous.

	Relative MM ($g^{1/3}$ of muscle/$kg^{1/3}$ body mass)		Relative PCSA (cm of muscle/$kg^{1/3}$ body mass)		Relative FL (cm of muscle/$kg^{1/3}$ body mass)	
	Clawed	Declawed	Clawed	Declawed	Clawed	Declawed
Total Forearm	1.71	1.82	2.43	2.58	1.02	1.02
Total Flexors	1.42	1.48	1.87	1.86	0.85	0.93
Total Extensors	1.07	1.17	1.12	1.24	1.04	1.07
Wrist Flexors	1.03	1.08	1.13	1.24	0.83	0.73
Digital Flexors	1.21	1.26	1.49	1.39	0.87	1.04
Wrist Extensors	0.86	1.04	0.89	1.05	0.79	1.06
Digital Extensors	0.85	0.79	0.67	0.66	1.20	1.07
FCR	0.70	0.62	0.67	0.59	0.71	0.65
FCU	0.87	0.78	0.82	0.78	0.9	0.74
PL	0.49	1.02	0.40	1.08	0.79	0.88
FDS	0.71	0.76	0.8	0.71	0.55	0.81
FDP	1.05	1.06	1.06	0.96	1.00	1.21
ADIL	0.53	0.70	0.57	0.70	0.37	0.68
ECU	0.66	0.77	0.63	0.78	0.67	0.71
ECRL	0.51	0.66	0.35	0.51	1.32	1.04
ECRB	0.61	0.70	0.52	0.45	0.77	1.55
BR	0.69	0.72	0.38	0.39	2.14	2.34
Sup.	0.48	0.51	0.61	0.69	0.31	0.27
PT	0.75	0.83	0.75	0.84	0.72	0.77
PQ	0.59	0.41	0.61	0.56	0.48	0.21

Supplemental Table S2. Relative leg muscle mass, PCSA and FL for functional groups and individual muscles for the declawed and intact kinkajous.

	Relative MM ($g^{1/3}$ of muscle/$kg^{1/3}$ body mass)		Relative PCSA (cm of muscle/$kg^{1/3}$ body mass)		Relative FL (cm of muscle/$kg^{1/3}$ body mass)	
	Clawed	Declawed	Clawed	Declawed	Clawed	Declawed
Total Leg	1.75	1.76	2.37	2.58	1.42	1.24
Plantar Flexors	1.58	1.60	1.80	2.01	1.20	1.02
Dorsiflexors	1.12	1.10	0.82	0.82	2.04	1.89
Non-Digital Plantar Flexors	1.26	1.24	1.30	1.39	1.15	0.95
Digital Flexors	1.03	1.11	1.00	1.21	1.1	1.05
Evertor	0.77	0.74	0.60	0.61	1.27	1.04
Invertors	1.12	1.12	0.82	0.89	2.03	1.69
Digital Extensors	0.68	0.70	0.44	0.42	1.64	1.91
Gas. M.	0.86	0.84	0.77	0.78	1.00	0.94
Gas. L.	0.89	0.80	0.69	0.69	1.39	1.01
Plant.	0.71	0.69	0.56	0.54	1.1	1.04
Sol.	0.87	0.71	0.77	0.66	1.07	0.81
FDIL	0.64	0.68	0.61	0.78	0.67	0.49
FDL	0.90	0.84	0.74	0.70	1.27	1.16
FDB	0.75	0.78	0.62	0.67	1.00	0.98
Fib. B.	0.64	0.54	0.47	0.37	1.12	1.08
Fib. L.	0.61	0.63	0.41	0.48	1.36	1.00
TCa	0.70	0.94	0.49	0.76	1.34	1.35
TCr	1.02	0.99	0.68	0.71	2.19	1.89
